# A Systematic Review of Interventions to Reduce Problematic Substance Use Among Transgender Individuals: A Call to Action

**DOI:** 10.1089/trgh.2016.0037

**Published:** 2017-03-01

**Authors:** Tiffany R. Glynn, Jacob J. van den Berg

**Affiliations:** ^1^ Department of Behavioral and Social Sciences, Brown University School of Public Health, Providence, Rhode Island.; ^2^ Department of Psychology, University of Miami, Coral Gables, Florida.; ^3^ Department of Medicine, The Warren Alpert Medical School of Brown University, Providence, Rhode Island.; ^4^ Division of Infectious Diseases, The Miriam Hospital, Providence, Rhode Island.

**Keywords:** alcohol, drug use, substance use interventions, substance use treatment, systematic review, transgender individuals

## Abstract

Persons who are transgender (i.e., individuals who are assigned one sex at birth, but who do not identify with that sex) are at elevated risk for developing problematic substance use. Recent studies indicate that transgender persons have high rates of alcohol use, illicit drug use, and nonmedical use of prescription drugs and evince more severe misuse of these substances compared with nontransgender individuals. Despite the high rates of substance use among transgender persons and the multiple conceptual and narrative recommendations for substance use treatments, there is a lack of consensus or awareness of empirically tested interventions and programs effective for this population. Thus, it is critical to examine current substance use interventions for transgender individuals to identify gaps in the field and to immediately put forth efforts to reduce problematic substance use. This systematic review is the first to attempt a comprehensive identification and synthesis of the available evidence on interventions for reducing problematic substance use among transgender persons. Reflective of the state of the field regarding transgender care for substance use, we found a deficiency of studies to include in this systematic review (*n*=2). Perhaps the most important conclusion of this review is that well-designed, theoretically informed culturally sensitive research focused on developing and rigorously testing interventions for substance use among transgender individuals is alarmingly scarce. This review discusses barriers to intervention design and synthesizes treatment recommendations for future work.

## Introduction

Persons who are transgender (i.e., individuals who are assigned one sex at birth, but who do not identify with that sex) are at elevated risk for developing problematic substance use.^1^ Problematic substance use can be considered the umbrella term to encompass the multiple facets and terminology used by the World Health Organization,^2^ the National Associations for National Institute on Alcohol Abuse and Alcoholism (NIAAA),^3^ and the National Institute on Drug Abuse^4^ for using alcohol or other drugs in excess. For example, engaging in binge drinking (e.g., defined as four to five or more drinks consumed on one occasion at least once in a 2-week period), using prescription medication(s) more than prescribed or not with a doctor's prescription, or regular drug use (e.g., opioids, amphetamines, or cocaine). It also encompasses experiencing substance-related problems (e.g., motor vehicle accident from driving under the influence) or having any substance use disorder diagnoses as defined by the Diagnostic and Statistical Manual of Mental Disorders.^5^ Recent studies indicate that transgender individuals have high rates of alcohol use (estimates up to 72%), marijuana (estimates up to 71%), other illicit drug use (estimates up to 34% [including intravenous drug use]), and nonmedical use of prescription drugs (estimates up to 26.5%) and evince more severe misuse of these substances compared with nontransgender persons.^6–14^

In examining this disparity between transgender and nontransgender individuals, studies have noted associations between trans-specific discrimination (e.g., transphobia, gender abuse, and religious abuse) and substance use.^15–20^ These findings support the Minority Stress Model,^21^ which posits that prolonged exposure to prejudice and discrimination experienced by members of minority and marginalized groups is associated with adverse psychological outcomes and health risk behaviors such as substance use.

Other scholars investigating problematic substance use among transgender populations have done so in the context of HIV risk due to the high prevalence of HIV among this group, especially transgender women, which has been well-documented across numerous studies.^22–24^ A syndemic framework^25^ has been used to contextualize the HIV epidemic among transgender persons positing that HIV risk is rooted in multiple co-occurring public health problems, including psychological issues, violence, discrimination, and substance use, which act synergistically to drive sexual risk behaviors.^24,26–29^ This syndemic among transgender communities can be conceptualized as intertwining epidemics (including problematic substance use) that act multiplicatively to mutually reinforce and predict HIV risk. For example, HIV-related risk behaviors (e.g., condomless sex) have been shown to be specifically exacerbated by substance use,^30^ especially among transgender communities,^12,15,31–35^ demonstrating that solely focusing on sexual behaviors for reducing the transmission of HIV may not be sufficient without also targeting problematic substance use. Given the high prevalence of problematic substance use among transgender persons, it is important for researchers, public health professionals, and policy makers to understand how problematic substance use contributes to this syndemic to intervene and reduce behavioral risks.^23,36^

In response to these substance use rates and the association with HIV, scholars dedicated to lesbian, gay, bisexual, and transgender (LGBT) health have called for substance use interventions specifically for transgender populations. More than 15 years ago, Lombardi and van Servellen^37^ proposed the need for specialized substance use treatments for LGBT people to remedy the deficiencies (e.g., discrimination, biases) found in general treatment programs. Following this, the Substance Abuse and Mental Health Services Administration (SAMHSA) released a policy acknowledging the special needs of these populations with the aim to make researchers and treatment providers aware of LGBT-specific issues in substance use treatment.^38^ Stevens^39^ furthered this call for specialty substance use treatment for LGBT individuals, but acknowledged the barrier that specialized care is difficult to find; thus, cultural sensitivity training for providers and staff in general care is still urgently needed today.

More specific to transgender individuals, the Transgender Substance Abuse Treatment Policy Group of the San Francisco Lesbian, Gay, Bisexual, and Transgender Task Force^40^ provided guidelines regarding cultural sensitivity and program design for substance use treatment programs to adopt in order to facilitate better care for transgender persons. The need for specialized programs designed to meet the unique needs of transgender individuals (e.g., addressing facets of gender affirmation, minority stress, or the syndemic framework regarding co-occurring problems) and cultural competency among treatment providers continues to be reiterated in the burgeoning literature in this area.^13,17,39,41,42^ Taken together, there are two paths to improve substance use intervention outcomes for transgender individuals: programs designed specifically for transgender persons and revamping general substance use programs to be sensitive to transgender needs within their more broad treatment.

Although this call for specific attention to address problematic substance use among transgender individuals has been made, there has been no systematic investigation into the current state of interventions for the transgender community. The majority of systematic reviews and meta-analyses examining substance use interventions broadly have focused on general adult or adolescent populations^43–49^ and have not been dedicated to specific subpopulations. This could be a reflection of the fact that evidence-based practices for problematic substance use are mainly developed and tested for the majority population. This general focus disregards the heterogeneity in specific subpopulations, especially for those at higher risk for problematic substance use such as those in the transgender community. Substance use programs that have been adapted or that have added elements to address issues specific to people's unique parameters (e.g., race/ethnicity, age, and sexual orientation) have been shown to be effective in helping facilitate harm reduction and reduced use of substances,^50–53^ indicating a need to utilize culturally sensitive interventions and programs when available.

Despite the high rates of substance use in transgender individuals and the multiple conceptual and narrative recommendations for substance use treatments, there is a lack of consensus or awareness of empirically tested interventions and programs effective for transgender persons. Given the political and social climate of the transgender community gaining more visibility and greater concentrated efforts promoting their health and civil rights, it is critical to review current substance use interventions for transgender individuals to identify gaps in the field and to immediately put forth efforts to reduce problematic substance use. Moreover, due to the multiplicative nature of HIV risk and substance use in transgender communities, a review would be beneficial because knowing the most effective ways of intervening on problematic substance use may reduce HIV incidence.

This is the first known systematic review investigating interventions for problematic substance use for transgender individuals. The aims of this study were to (1) explore the literature for substance use interventions for transgender individuals in order to examine the current state of the field; (2) describe the characteristics of included studies; (3) summarize the findings across included studies to examine efficacy and inform future work; and (4) synthesize treatment recommendations from the literature to advise intervention development.

## Methods

### Study selection

This review followed an *a priori* systematic review protocol to reduce bias of selection and reporting of studies. Our *a priori* protocol identified search parameters, including the population of interest, the types of interventions to include, if interventions needed to have any control or comparison group, and defining the outcomes of interest.

#### Population

For population eligibility, included studies were those that targeted intervention activities specifically toward transgender people. Studies that targeted transgender individuals in addition to other populations were included if they disaggregated findings for transgender participants. Included transgender populations were not restricted to the United States and could be from any country as long as there was an English-language translation of the article.

#### Intervention

To truly examine all available interventions in the literature, any type of study design evaluating an intervention for reducing problematic substance use was included. Eligible interventions could take a psychosocial, behavioral, structural, or medical approach and could also address other comorbid issues, so long as the intervention had a clear component addressing substance use specifically.

#### Comparison group

To ensure greatest sensitivity, any type of comparison group or those with no comparison group were included.

#### Outcomes

Problem substance use was interpreted broadly and included studies designed to target those using substances beyond recommended and/or medical guidelines (e.g., weekly drinking levels as defined by the NIAAA; using prescription medication more than prescribed or not with a doctor's prescription), those experiencing substance-related problems, or those with a substance use disorder diagnoses. The primary outcomes of interest in this review were changes in substance use, as measured by biological markers or self-report.

### Search strategy

**FIG. 1.**
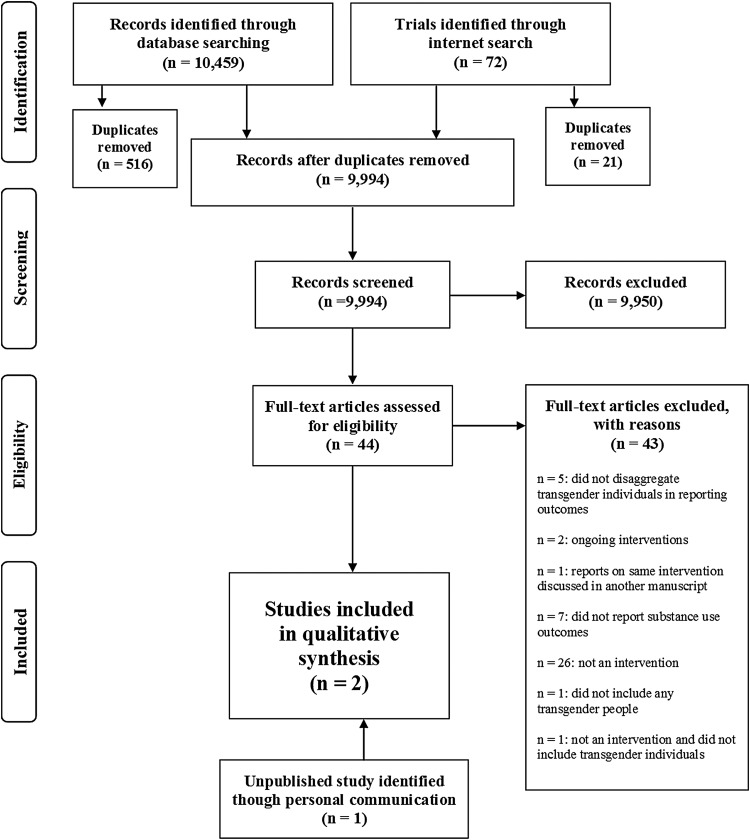
Preferred Reporting Items for Systematic Reviews and Meta-Analyses (PRISMA) flowchart of systematic review.

Five electronic databases were searched (PubMed, PsychINFO, LGBT Life, CINHAL, and CENTRAL) for studies published through July 2016 (see Fig. 1 for the Preferred Reporting Items for Systematic Reviews and Meta-Analyses [PRISMA]^54^ flowchart of systematic review). The search strategy included free-text terms relating to populations and outcomes of interest to be able to use the same search across databases. The search terms for the electronic databases for the outcome of interest, changes in substance use, were derived from past Cochrane Reviews,^55,56^ illicit drugs measured in the Structured Clinical Interview for Diagnostic and Statistical Manual of Mental Disorders (DSM),^57^ and a past review regarding alcohol interventions.^58^ To specify the population of interest, we used search terms for transgender individuals derived from a past review^59^ and also additional terms derived from consultation with a published and experienced researcher in the field of transgender health. To increase sensitivity and given the wide criteria for any type of intervention and any or no comparison group, no search filters were used for intervention type (see Appendix A1 for all search terms). In addition to the electronic databases, ongoing and completed clinical trials were searched through Internet searches (see Appendix A2 for included websites and search terms). This was done to capture any completed clinical trial not found in the database search and to also be able to find and report on any current interventions being conducted. Although ongoing interventions cannot be included in the review due to lack of outcomes, acknowledging this work adds to the understanding of the current state of the field. The results of the five sets of electronic searches were collated (*n*=10,459) into a single database and duplicates removed. Internet searches were conducted separately (*n*=72) and duplicates removed. Titles and abstracts of all identified records after duplication removal (*n*=9,994) were initially screened by the first author to exclude citations that were not relevant. A total of 44 records met preliminary inclusion criteria based on information presented in the abstract or title. Full texts for all preliminary eligible records were retrieved, which were then thoroughly read for further eligibility separately by both authors and then discussed to reach a consensus based on the *a priori* criteria. This produced the final list of included studies (*n*=1). Due to the lack of studies found, and to produce a thorough review, academic experts in transgender health were contacted (*n*=10) and asked to add any additional studies they were aware of. Five experts responded and this personal communication produced one more intervention to include in the current review that has been presented at a national conference with the primary outcome article in preparation, thus making the final list of included studies *n*=2.

### Data extraction

For the included studies, data were extracted by the first author and included details about study participants, inclusion criteria, intervention design, and results (Table 1).

**Table 1.** **Summary of Characteristics of Included Study and Main Findings**

Study	Participants	Inclusion criteria	Intervention	Intervention length	Main findings
Nemoto et al.^45^	Between October 2001 and September 2003, a total of 359 transgender women were eligible for the program; 206 enrolled in the program and 109 completed the full program and provided post-test data. Study examined data from 109 who completed it.	Transgender women who reside or work in San Francisco aged 18 years or older	“Transgender Resources and Neighborhood Space (TRANS) program”	Between 10 and 18 workshops; those attending 10 of the 18 were eligible to graduate from the program	1. Significant reductions in perceived barriers to substance abuse treatment programs 2. Marginal reductions in alcohol use during the past 30 days 3. No changes in illicit drug use
			1-hour group workshops organized around three domains:		
			1. Sex, relationships, and health		
			2. Reducing drug use and improving coping skills		
			3. General life needs		
			Conducted by transgender women health educators in both English and Spanish.		
			Health educators use multiple group facilitation techniques:		
			1. Interactive discussions		
			2. Personal expression exercises		
			3. Videos and media		
			4. Guest lectures		
Nemoto et al.^61^	Between March 2011 and August 2012, a total of 114 transgender women of color enrolled in the study and completed the intake session. At the 3-month follow-up, 93 women completed the assessments and 78 women completed the 6-month follow-up.	African American or Latina, transgender woman, aged 18 years or older, working or residing in San Francisco area	“Transgender Empowerment and Motivational Interviewing (TEAM-I)”	All participants completed: 1. intake assessment 2. 3-month assessment 3. 6-month assessment Additionally, MEI group: completed 3 MI sessions between intake and 3 months and 3 MI sessions between 3 months and 6 months; BI group: 1 BI session at intake and 1 BI session at 3 months	Alcohol, marijuana, and amphetamine usage decreased among all intervention groups from intake to 6-month follow-up; however, the MEI group showed highly significant (*p*≤0.01) decreases when compared with the BI and Control groups MEI intervention participants showed the most significant decreases in alcohol, marijuana, and amphetamine use frequency at 6-month follow-up, when compared with intake frequencies.
			Participants were randomly assigned to one of the following groups:		
			1. MEI		
			• Culturally sensitive intervention curriculum was developed using community-based participatory research		
		Current substance user (drinking alcohol almost every day or using drugs more than 3 days a week in the past 6 months)	• MI based on the idea of stages of change		
			• Objectives		
			➢(1) Reduce substance use; (2) Reduce sexual risk; (3) Develop supportive social networks and engage in healthier/prosocial community activities; (4) Increase self-esteem and pride in being transgender women		
			2. BI		
			• Brief individualized health promotion education		
			• Objectives		
			➢(1) Provide brief information and guidance about substance abuse and HIV risk reduction; (2) Provide information about hazardous substance use and HIV risk behavioral patterns (i.e., differences between ideal and actual behaviors) without confrontation and apprise the positive intention or ideas for substance abuse and HIV prevention; (3) Present a menu of options for prevention and/or behavioral change methods and plan the achievable short-term goals		
			3. Control group		
			• Provided with substance abuse and HIV prevention information		
			Each intervention session took about 1.5–2 hours		
			Conducted by African American and Latina (transgender women) health educators in both English and Spanish.		

BI, brief intervention; MEI, motivational enhancement intervention; MI, motivational interviewing.

## Results

The majority of studies reviewed were not an intervention (*n*=26), and others discussed substance use interventions for transgender individuals, but did not report any outcomes (*n*=7). There were some studies that identified transgender individuals in their samples, but did not report separate outcomes for them (*n*=5), so these studies were excluded. Another study (*n*=1) did not include any transgender people, and another (*n*=1) was not an intervention nor did it include transgender people. One article discussed an intervention that was already reported on in another reviewed article (see Table 2 for summary of exclusions). Although excluded, many of these studies had great value in informing the discussion of the results. Two ongoing interventions were found (Table 3), so effectiveness cannot be currently discussed, but these should be acknowledged to examine outcomes in the future.

**Table 2.** **Reviewed Full-Texts and Reasons for Exclusion**

Author	Reasons excluded
Brown et al.^70^	Not an intervention
Dunckley^86^	Not an intervention
Finlon^87^	Not an intervention
Gelaude et al.^88^	Not an intervention
Gilbert et al.^89^	Not an intervention
Heck et al.^90^	Not an intervention
Hellman et al.^91^	Not an intervention
Jordan^92^	Not an intervention
Keuroghlian et al.^65^	Not an intervention
Klein and Ross^93^	Not an intervention
Krishnan et al.^94^	Not an intervention
Lyons et al.^71^	Not an intervention
Medeiros et al.^95^	Not an intervention
Nuttbrook et al.^84^	Not an intervention
Patel et al.^96^	Not an intervention
Pettifor et al.^97^	Not an intervention
Polak et al.^81^	Not an intervention
Ritter^98^	Not an intervention
Senreich^99^	Not an intervention
Senreich^100^	Not an intervention
Senreich^72^	Not an intervention
Silvestre^101^	Not an intervention
Stevens^39^	Not an intervention
Warren et al.^102^	Not an intervention
Wolf et al.^13^	Not an intervention
Ziegler^103^	Not an intervention
Goldbach et al.^104^	Did not report substance use outcomes
Goyal et al.^105^	Did not report substance use outcomes
Hicks^106^	Did not report substance use outcomes
Maguen et al.^107^	Did not report substance use outcomes
Oggins^76^	Did not report substance use outcomes
Reback et al.^24^	Did not report substance use outcomes
Rebeck et al.^108^	Did not report substance use outcomes
Harris et al.^79^	Did not report separate outcomes for transgender individuals
Maxwell^109^	Did not report separate outcomes for transgender individuals
Rowan^110^	Did not report separate outcomes for transgender individuals
Schwinn et al.^111^	Did not report separate outcomes for transgender individuals
Yu et al.^112^	Did not report separate outcomes for transgender individuals
Senreich^113^	Did not include transgender individuals
Allison et al.^114^	Not an intervention, nor did it include transgender individuals
Nemoto et al.^78^	Intervention was already reported on in another reviewed article^60^

**Table 3.** **Ongoing Substance Use Interventions for Transgender Individuals**

Principal investigator	Title	Purpose	Participants	Intervention	Estimated primary completion date	Primary outcome measures
Jeffrey T. Parsons	Multicomponent Intervention to Reduce Sexual Risk and Substance Use	Expand and refine an intervention for transgender women into a 7-session individual- and group-based intervention that is scalable for community settings to reduce sexual risk and substance use and to increase stigma-coping and risk-buffering behaviors.	1. 18 years of age or older	MI+CBST	January 2017	1. Decreases in the number of unprotected sex acts
			2. Identify as a transgender woman	The intervention focuses on exploring health goals, creating an action plan, learning about the impact stress, stigma, and substance use can have on health, improving personal growth and social support, and connecting with resources.		2. Decreases in the number of days of drug use
Robert Garofalo Niranjan Karnik	Employing eSBI in HIV Testing Environments for At-Risk Youth	Assess the feasibility and acceptability and test the initial efficacy of eSBI (eSBI for alcohol use) coupled with STTR in comparison with STTR only among YMSM and young transgender women on frequency of substance use and engagement within the HIV and PrEP care continuum.	1. Aged 16–25 years	eSBI for alcohol use	March 2019	1. Change in frequency of alcohol use
			2. Self-identify (or are behaviorally) as MSM or as a transgender woman	Those who are randomized to the intervention will complete eSBI for substance use, which comprises 11 topical areas, each with a single webpage, in an MI format.		2. PrEP and HIV-related medical care engagement (completed clinical visits)

See ClinicalTrials.gov for details of each study.

CBST, cognitive-behavioral skills training; eSBI, electronic screening and brief intervention; MI, motivational interviewing; PrEP, pre-exposure prophylaxis; STTR, seek, test, treat, and retain; YMSM, young men who have sex with men.

### Characteristics of included studies

Of the 44 studies screened for inclusion and after personal communication with academic transgender health experts, one cohort study published in 2005^60^ and one presented at a national conference and unpublished randomized control trial (RCT)^61^ were selected for this systematic review, reporting data from 109 transgender participants and 114 transgender participants, respectively (Table 1).

### Intervention design and content

Both the Transgender Resources and Neighborhood Space (TRANS) program and the Transgender Empowerment and Motivational Interviewing (TEAM-I) study were studies from the same research group and aimed to reduce HIV risk and substance use among transgender women in San Francisco, with the latter focusing on African American women and Latinas.

#### TRANS program

The intervention was designed as 18 one-hour group workshops, held weekly, organized into three domains: (1) sex, relationships, and health; (2) reducing drug use and improving coping skills; and (3) general life needs. Individuals were eligible to be in this study if they were transgender women aged 18 years or older residing or working in San Francisco. These sessions were conducted by health educators (all of whom were transgender women) in both English and Spanish. The health educators used multiple group facilitation techniques such as interactive discussions, personal expression exercise, videos and media, and guest lectures. The transgender women were encouraged to finish the workshops at their own pace, as opposed to following a strict schedule (average time to complete 10 sessions was 6 weeks). Nemoto et al.^60^ also noted that the women were encouraged to socialize at the TRANS program site, which provided a living room area and shower facility. A resource closet with donated clothing and accessories free to those in need was also provided to create a safe and culturally sensitive space for participants to engage with the intervention. Participants were asked to attend at least 10 of the 18 workshops to be considered program graduates. Preintervention, the women were asked to complete assessments on sexual behaviors, substance use, attitudes toward substance abuse treatment, HIV knowledge, depression, self-esteem, and transgender community involvement. If considered a graduate of the program (i.e., completing at least 10 workshops), participants were then asked to complete postintervention assessments of the same outcomes within 2 weeks of completion.

#### TEAM-I

This intervention was designed as an RCT specifically for transgender African American women and Latinas using three group conditions: (1) motivational enhancement intervention (MEI); (2) brief individualized health promotion education (BI); and (3) the control condition. Eligibility criteria included 18 years or older, African American or Latina, transgender woman, living or working in the San Francisco area, and reported drinking alcohol almost every day or using drugs more than 3 days a week in the past 6 months. All participants completed outcome assessments at an intake session, a 3-month follow-up session, and a final 6-month follow-up session. Additionally, the MEI group completed one MEI session at intake and two more before the 3-month assessment, then one at the 3-month assessment, and the final two MEI sessions before the 6-month assessment. The BI group completed one BI session at intake and one BI session at the 3-month session. The control group was asked to only complete the assessment sessions. The MEI protocol and aims were designed using community-based participatory research with a community advisory board comprising individuals from the San Francisco Department of Public Health, community-based organizations serving the transgender community, and transgender community members (see Table 1 for details about each condition). As in the other included study, these sessions were conducted by health educators (all of whom were transgender women) in both English and Spanish. However, in contrast to the TRANS workshops delivered in groups, all sessions were delivered individually.

### Summary of intervention treatment effects

#### TRANS program

Participant baseline levels of any alcohol use and any illicit drug use were 57.3% and 46.3%, respectively. In regard to substance use outcome differences between pre- and postassessment, findings indicated significant reductions in perceived barriers to substance abuse treatment programs (*p*=0.001); marginal reductions in alcohol use during the past 30 days (*p*=0.06); and no changes in illicit drug use. The authors of the intervention noted that perhaps the marginally significant results would have been significant if a longer time lag had been implemented for administering follow-up surveys. In addition, longer time before follow-up might have allowed for more noteworthy behavioral changes in substance use. Another limitation to evaluating treatment effects was the lack of rigorous testing (i.e., no randomization, no control group). Furthermore, women were not screened in on the basis of their substance use, thus a major limitation in evaluating treatment effects.

#### TEAM-I

Findings indicated that alcohol, marijuana, and amphetamine usage decreased among all intervention groups from intake to 6-month follow-up; however, the MEI group showed highly significant (*p*≤0.01) decreases when compared with the BI and control groups. The MEI intervention participants showed the most significant decreases in alcohol, marijuana, and amphetamine use frequency at 6-month follow-up, when compared with intake frequencies.

## Discussion

This systematic review is the first to attempt a comprehensive identification and synthesis of the available evidence on interventions for reducing problematic substance use among transgender individuals. This review has clearly identified a large gap in the field of transgender health that needs attention and should be used as a call to action for efforts to address problematic substance use. The findings of this review elicit questions regarding how this major gap in the research literature has been facilitated and how to remedy it moving forward. In summary, there has been a lack of inclusion of transgender individuals in the current substance use research and intervention work; studies that do include transgender individuals have not collected gender identity or have not disaggregated findings to examine outcomes separately; and there is a lack of interventions developed specifically for transgender persons.

Perhaps one trend facilitating the gap between the need for treatment specific to transgender individuals and actual programs offering this care is the false belief that treatments do exist. Cochran et al.^62^ investigated if specialized substance use services existed for LGBT persons and found that of the 911 treatment listings in the United States and Puerto Rico, 11.8% of substance use programs indicated that they offer specialized services for sexual and gender minorities. However, the researchers conducted phone surveys and they revealed that 70.8% of these programs are the same services offered to the general population, and only 7.4% identified a service specifically tailored to the needs of LGBT individuals, which calculates to only eight programs of 911 substance use programs throughout the United States and Puerto Rico. False advertisement of treatment services that do claim specialized programs propagates the perception of having more services than are actually available and masks the real need for intervention development.

In addition, facilitating this gap is the issue that gender identity is rarely collected in substance use research, which is the most fundamental step to begin to address substance use intervention work. Between 2010 and 2011, the Institute of Medicine and US Department of Health and Human Services both set forth agendas to prioritize efforts for LGBT health research.^1,63^ Flentje et al.^64^ examined the reporting of sexual orientation and gender identity in the substance use literature during a recent time period before these national agendas were set forth (i.e., 2007). They then repeated this for a time period after these agendas (i.e., 2012) to see if the call for LGBT health research by national agencies moved the substance use field forward into efforts examining LGBT individuals. They found that in 2007, reporting of gender beyond the binary (i.e., male and female only) in the substance use literature happened between 0% and 1% in 2007 and was only raised to between 1.9% and 2.3% in 2012. The omission of collecting gender identity in substance research is a missed opportunity to not only understand the well-being of transgender people, but it also means we are not learning about sex and gender differences that may be relevant to all people.

Beyond collecting gender identity, recruitment of gender minorities in substance use treatment studies is also crucial to be able to examine outcomes for these populations. For the literature that does collect and report on gender identity, it is commonly collapsed with sexual orientation, which is problematic because it prevents conclusions about the impact of substance use interventions for transgender individuals. For instance, five of the studies in this systematic review were excluded due to not reporting substance use outcomes separately for their transgender participants. There is a need to disaggregate findings for the T in LGBT, and also from more general populations, to develop effective evidence-based interventions specifically developed for transgender persons. Perhaps collapsing these groups together stems from the small number of transgender individuals within substance use intervention studies, pointing to the need for concentrated efforts in recruiting transgender individuals to be included within these studies.

In considering the way to move forward to address the identified gap of this review, it is critical to examine the body of research on substance use treatment for transgender individuals identified during this review, but excluded for methodological criteria. This literature has examined past experiences, barriers, acceptability, and associations of attending treatment.^6,37,39,62,65–72^ Important considerations from this research have emerged for future substance use intervention design. Numerous barriers for transgender individuals seeking treatment have been found that should be addressed when designing substance use programs, including unknowledgeable personnel in substance use treatments on trans-specific realities and experiences, treatment providers having negative attitudes toward transgender individuals, victimization (e.g., verbal, physical, and sexual abuse by other clients and staff), discrimination (e.g., being required to wear only clothes judged to be appropriate for their birth sex), and little formal education for staff regarding the needs of transgender people.^13,37,68,71–76^ Additionally, the current literature provides consistent themes in treatment recommendations for trans-specific substance use programs (Table 4).

**Table 4.** **Overarching Themes in Recommendations for Substance Use Programs for Transgender Individuals**

Specialized transgender substance use programs
▸ Use gender minority theoretical frameworks and theories to identify specific issues that affect transgender individuals and then target them when developing interventions.
▸ Community-based participatory intervention design methods can help identify salient issues that need to be addressed specific to the transgender community.
▸ Multicomponent interventions that address co-occurring issues are warranted.
▸ Interventions should be delivered by transgender peers and should promote a positive identification with the transgender community.
General substance use programs
▸ Integrated care should be developed and amended to be culturally sensitive. Programs that are not trans specialized should make every effort to foster an environment and treatment experience of affirmation and inclusivity to allow for a transgender individual to focus on their problematic substance use.

One consistent recommendation for substance use interventions for transgender individuals is that using gender minority theoretical frameworks and theories (e.g., queer theory, transgender theory, and gender affirmation framework) can aid in identifying issues that affect transgender individuals specifically when developing interventions for substance use.^39^ Hendricks and Testa^77^ contextualized the Minority Stress Model for transgender individuals and recommend that the unique issues (e.g., transphobia, discrimination, and lack of support) that place transgender individuals at risk for health risk behaviors should be addressed directly in treatment. Others have also called for this theoretical approach in regard to substance use treatment for transgender individuals.^13,39,41^ Gender minority frameworks and theories can be supplemented by using community-based participatory research methods, as seen in the included intervention (TEAM-I). This would allow members and advocates of the community to confirm the needs of the transgender community that are posited by theories and frameworks and it may elicit new needs that have not been identified in the research.

A guideline endorsed by multiple scholars, and supported by the two included studies in this review, is that interventions should be delivered by transgender peers to allow for greater comfort in treatment and a more nuanced understanding of clients due to the unique experiences of transgender individuals.^60,76,78^ On a similar note, Hendricks and Testa^77^ suggest that clinicians should focus on promoting a positive identification with the transgender community as a means of buffering the negative effects of minority stressors, which is supported by the included studies in this systematic review. The Transgender Recovery Program has successfully implemented this within their residential substance use treatment program for transgender women.^76^ This intervention did not meet criteria for inclusion in the current review due to a lack of reporting substance use outcomes; however, they did report 81% retention in the program, noted as good compared with their general population program's retention of 60%. Clients are encouraged to develop peer supports through their assigned Big Sister (i.e., another transgender woman) and through weekly small group meetings. The included study in this review also incorporated a community networking and empowerment domain within the intervention and also encouraged the women to socialize with each other in the program site. This group-level support provides a positive peer and community interaction that can foster resilience and recovery.

Addressing syndemic theory by building multicomponent interventions that address co-occurring issues is also warranted.^13,26,60,65,78,79^ Targeting sexual behaviors is important for reducing the transmission of HIV and other sexually transmitted infections, but intervening on these alone may be insufficient in reducing new infections. The Positive Steps Program for women (both cisgender female and female identified) reported successful reductions in problematic substance use while targeting multiple facets of the HIV syndemic.^79^ This program did not meet criteria to be included in this review due to not separately reporting outcomes for transgender women, but warrants acknowledgement. This program is a 6-week residential chemical dependency program targeting substance use, sexual risk behaviors, and other person-level risk factors for HIV (e.g., gender identity and discrimination), which showed that alcohol and drug use declines from baseline to exit of program and to the 12-month follow-up along with a decline in psychological distress and sexual risk behaviors (i.e., condomless sex with an HIV-positive partner). The included programs, the TRANS project and TEAM-I, aimed to address multiple issues for transgender women such as HIV, drug use, discrimination, job seeking, relationships, and community involvement. By addressing the various epidemics that act multiplicatively to reinforce each other and facilitate substance use, it provides opportunity for long-term positive health outcomes and more holistic well-being.

Although specialized programs seem to be needed the most, there should be a greater awareness that these may not be easily and widely implemented due to various reasons such as the climate of transgender acceptance in some geographic locations^80^ or lack of financial resources to support these types of specialized programs. We should not only consider specialized programs but also substance use treatment providers should be challenged to meet the specific needs of transgender substance users within mainstream clinical settings to reduce further stigmatization and marginalization.^39^ Within these mainstream clinical settings, providers should be culturally competent to meet the needs of transgender people.^13,81^ Such integrated care should also be amended and developed to be culturally sensitive to transgender persons. For instance, although some trans-specific issues may be intertwined in an individual's substance use, providers need to be educated about identifying when gender issues are peripheral and not relevant to substance use treatment as to not overstate the entire transgender experience as a risk factor.^82^

Preventing discrimination by healthcare providers is also crucial for optimal treatment outcomes. Not specific to transgender individuals, negative attitudes have been found among healthcare professionals toward persons with problematic substance use and these attitudes have been associated with substandard treatment and care.^83^ Additionally, substance use treatment providers have been shown to have the least education about transgender individuals and have the greatest negativity toward them.^68^ Collectively, transgender individuals needing substance use treatment face exacerbated discrimination by providers for not only having problematic substance use but also for being transgender. Preventing discrimination within general substance use care from occurring produces a greater likelihood of successful treatment. Treatment staff should also be trained in transgender competence and sensitivity to make sure transgender inclusivity is not just written in a program's mission, but actually being practiced.^41^

Awareness of the unique needs of transgender people and sensitivity toward them are important in affirming an individual and providing an environment of acceptance. Examples include not restricting individuals' access to restrooms that are appropriate for their gender identity, not conflating transgender individuals with sexual minorities (lesbian, gay, etc.), allowing for gender presentation resources (e.g., makeup and clothing), sleeping arrangements or housing according to gender identity, allowance of hormone use, and using proper pronouns.^37,72,76,84^ In a sample of transgender individuals who had been in residential treatment for substance use, individuals who felt their environment was inclusive had more positive treatment experiences compared with those experiencing stigma and who left treatment prematurely.^71^ Programs that are not trans specialized should make every effort to foster an environment and treatment experience of affirmation and inclusivity to allow for a transgender individual to focus on their problematic substance use.

Limitations in the field should be noted when considering our findings. There may be transgender-specific substance use programs that are effective, but have not been integrated into the literature if they were not implemented as part of research. Researchers should consider examining current community programs to rigorously evaluate them to add them to the body of evidence. Notably, the literature regarding transgender individuals has been historically rooted in HIV prevention; given this, there have been several HIV prevention interventions for transgender individuals. These interventions may have had domains peripherally targeting substance use, but were not reported on due to the study's emphasis on HIV outcomes. The paucity of included studies only allowed for a collapsed examination of interventions for transgender women and transgender men. It is important to note that these groups have distinct experiences and may need disparate interventions. Both included studies were focused on transgender women, reflecting the lack of inclusion of transgender men in the current literature regarding transgender persons. Little is known about the substance use behaviors and risks associated with such use for transgender men; given this, there has been a recent call for action to include transgender men in research.^11^

This systematic review was only able to synthesize overall treatment recommendations, but it should be acknowledged that the transgender community is heterogeneous with subgroups having different intervention needs (e.g., transgender people of color). It should also be noted that there is a spectrum of gender identities (e.g., gender nonconforming and gender queer) and these individuals should not be collapsed in with transgender substance use research. Although some overlapping experiences occur given their gender minority status, these other groups also need specific attentions. At the level of systematic review, a limitation of this study is that there could be unidentified literature reporting on substance use intervention outcomes if outside of the chosen search strategy. Limitations notwithstanding, this review identifies major gaps that need immediate attention to move the transgender health field forward.

## Conclusions

Although the treatment and intervention recommendations in the literature are important aspects of substance use research examining transgender individuals, moving toward designing and implementing culturally sensitive interventions are warranted and desired by the transgender community.^17^ Most recently, the NIAAA put forth an initiative to expand existing substance use research to focus on sexual and gender minority health^85^; this research agenda comes at a time of great need. Given this current sociopolitical climate of promoting psychosocial health among LGBT individuals,^1^ this offers a prime window of opportunity to research efficacious interventions toward problematic substance use and its psychological and social sequelae. Perhaps the most important conclusion of this review is that well-designed, theoretically informed culturally sensitive research focused on developing and rigorously testing interventions for substance use among transgender individuals is alarmingly scarce. The research regarding predictors, associations, barriers, and needs for transgender substance use treatment has been well documented; it is now time to design, implement, and disseminate interventions using this information to provide needed services to the transgender community.

## Appendix

**Appendix A1.** **Search Terms for Electronic Databases**

Population: transgender
transgender OR transgend^*^ OR trans-gend^*^ OR transsex^*^ OR trans-sex^*^ OR transex^*^ OR “sex reassign^*^” OR “sex change” OR “gender reassign^*^” OR “gender change” OR intersex^*^ OR “gender identity disorder” OR “MTF” OR “male to female transgender” OR “male-to-female transgender” OR “male to female transsexual” OR “male-to-female transsexual” OR “male to female transexual” OR “male-to-female transexual” OR “FTM” or “female to male transgender” OR “female-to-male transgender” OR “female to male transsexual” OR “female-to-male transsexual” OR “female to male transexual” OR “female-to-male transexual” OR transvest^*^ OR “travesty” OR “koti” OR “kothi^*^” OR “hijra” OR “mahuvahine” OR “mahu” OR “waria” OR “banci” OR “calabai” OR “kedie” OR “kawekawe” OR “wan du” OR “bissu” OR “katoey” OR “kathoey” OR “pet tee sam” OR “phuying praphet song” OR “sao praphet song” OR “nang fa jam leng” OR “ork-sao” OR “tut” OR “ladyboy” OR “ladyman” OR “meti” OR “cross dress^*^” OR “acault” OR “bayot” OR “bantut” OR “bakla” OR “bian xing ren” OR “bong lo” OR “nadleehi” OR “berdache” OR “xanith” OR genderqueer OR “gender queer” OR “gender nonconforming” OR “gender non-conforming”

**Outcome: substance use**
“substance use disorder” OR “alcohol use disorder” OR “substance use disorders” OR “alcohol use disorders” OR misuse OR dependen^*^ OR abuse OR substance OR polysubstance OR drug^*^ OR polydrug OR illicit OR illegal OR abstin^*^ OR abstain^*^ OR abuse^*^ OR addict^*^ OR overdose OR withdrawal^*^ OR disorder^*^ OR cocaine OR crack OR speed OR heroin OR amphetamine^*^ OR “designer drugs” OR narcotic^*^ OR “designer drug” OR methamphetamine^*^ OR crystal OR meth OR “off-label use” OR prescription OR opioid^*^ OR opiate^*^ OR opium OR ecstasy OR mdma OR hallucinogen^*^ OR lsd OR pcp OR ketamine OR acid OR “club drugs” OR “club drug” OR downer^*^ OR sedative^*^ OR barbiturate^*^ OR upper^*^ OR methadone OR morphine OR marijuana OR marihuana OR cannabis OR weed OR pot OR “hash oil^*^” OR hashish OR alcohol OR drink^*^ OR binge OR intoxic^*^

**Appendix A2.** **Search Strategies for Ongoing Studies**

Resource URL	Search terms
ClinicalTrials.govhttp://clinicaltrials.gov	Transgender OR transsexual
European Union Clinical Trials Register www.clinicaltrialsregister.eu	Transgender OR transsexual
World Health Organization International Clinical Trials Registry Platform Search Portal http://apps.who.int/trialsearch	Transgender OR transsexual

## Abbreviations Used

BI: brief intervention
CBST: cognitive-behavioral skills training
eSBI: electronic screening and brief intervention
LGBT: lesbian, gay, bisexual, and transgender
MEI: motivational enhancement intervention
MI: motivational interviewing
NIAAA: National Institute on Alcohol Abuse and Alcoholism
NIH: National Institutes of Health
PrEP: pre-exposure prophylaxis
RCT: randomized control trial
STTR: seek, test, treat, and retain
TEAM-I: Transgender Empowerment and Motivational Interviewing
TRANS: Transgender Resources and Neighborhood Space
YMSM: young men who have sex with men
